# Study on the Dynamic Changes in Fungal Communities During the Storage of Polygalae Radix and the Antifungal Effects of Peppermint Essential Oil

**DOI:** 10.3390/toxins17120585

**Published:** 2025-12-06

**Authors:** Hui Zhang, Yuying Su, Xinnan Wang, Ying Ren, Jinfeng Li, Jianping Han

**Affiliations:** Institute of Medicinal Plant Development, Chinese Academy of Medical Sciences & Peking Union Medical College, Beijing 100193, China

**Keywords:** Polygalae Radix, storage, fungal community, aflatoxins, essential oil, antifungal activity

## Abstract

Polygalae Radix, a traditional Chinese medicine for insomnia and memory disorders, is highly susceptible to fungal contamination and mycotoxin production (especially by *Aspergillus flavus*) during storage, compromising its safety and efficacy. Therefore, in this study, high-throughput sequencing was employed to evaluate the dynamic changes in fungal communities during the storage of Polygalae Radix and to analyze common mycotoxin-producing genera. Furthermore, the inhibitory effects of peppermint essential oil (PEO) on *A. flavus* were assessed through fumigation treatments, combined with colony counting and quantification of aflatoxins. Results showed the following: (1) Storage for 1–3 months significantly altered the fungal structure, promoting saprophytic and pathogenic fungi (e.g., *Wallemia*, *Paraphoma*, *Didymella*, *Cladosporium*…) and increasing the relative abundance of mycotoxin producers like *Penicillium*, *Aspergillus*, and *Fusarium* (notably, *Penicillium* increased from 0.28–2.33% to 5.39–80.43%). Additionally, *A. flavus*, capable of producing aflatoxins, was detected in samples stored for two months (RM2). (2) Antifungal tests demonstrated that PEO significantly inhibited the common fungi in Polygalae Radix. At 10 μL/g, it suppressed fungal growth and significantly reduced aflatoxin B_1_ (AFB_1_) and total aflatoxins (AFT, including AFB_1_, AFB_2_, AFG_1_, and AFG_2_) levels (*p* < 0.05). At 10 μL/g, AFB_1_ and AFT were reduced to undetectable levels. PEO can serve as a green and effective protective strategy to inhibit *A. flavus* during the storage of Polygalae Radix and control aflatoxin contamination.

## 1. Introduction

Polygalae Radix refers to the dried root of *Polygala tenuifolia* Willd. or *Polygala sibirica* L., which is widely used in traditional Chinese medicine for its anxiolytic, nootropic, expectorant, and anti-inflammatory activities and is used clinically for insomnia, amnesia, and other disorders [1]. Beyond medicinal use, Polygalae Radix is also valued for its nutritional and health-promoting properties, being incorporated into functional foods and soup bases, which contributes to its growing global demand [2].

Despite its wide utilization, Polygalae Radix is highly susceptible to fungal contamination, particularly by toxigenic species of the genus *Aspergillus*, during harvesting, processing, and storage. Poor ventilation, high humidity, and prolonged transportation markedly accelerate mold colonization and mycotoxin accumulation, jeopardizing product quality and medication safety [3]. Traditional storage practices, such as sealing freshly harvested roots in plastic bags to prevent weight loss, paradoxically create favorable conditions for the growth and toxin production of *Aspergillus flavus* (*A. flavus*). Moreover, water spraying treatment applied to facilitate core removal before acquisition by pharmaceutical manufacturers further increases the infection risk of toxigenic fungi.

Aflatoxins, secondary metabolites produced mainly by *Aspergillus* section *Flavi*, represent the most toxic and hepatocarcinogenic natural compounds identified to date [4,5]. Among them, aflatoxin B_1_ (AFB_1_) is classified as a Group 1 carcinogen by the International Agency for Research on Cancer (IARC) [6]. Notably, aflatoxins exhibit remarkable thermal and chemical stability, making them difficult to eliminate through conventional processing. In Polygalae Radix, the risk is particularly pronounced because the woody root core must be manually removed, leaving the peripheral medicinal tissue vulnerable to fungal invasion. According to the Chinese Pharmacopoeia (2020) [7], aflatoxin testing is mandatory for 16 herbal materials including Polygalae Radix, with maximum limits of 10 μg/kg for total aflatoxins (AFT, including AFB_1_, AFB_2_, AFG_1_, and AFG_2_) and 5 μg/kg for AFB_1_. Nevertheless, market surveys have reported an aflatoxin detection rate of up to 48% in Polygalae Radix, with an unqualified rate of 12% [8]. Post-harvest contamination has been directly linked to aflatoxicosis outbreaks [3], highlighting the need for stricter monitoring and safer storage practices.

Currently, research on the dynamic changes in fungal communities during Polygalae Radix storage remains limited. The colonization patterns of toxin-producing fungi and the succession of dominant fungal species are still unclear. Notably, mycotoxins can accumulate rapidly in favorable environments, and their occurrence in medicinal materials is closely associated with critical time windows and the activity of key microorganisms. Recent studies have demonstrated that the relative abundance of toxigenic fungi is a key determinant of toxin levels, making it a critical indicator for contamination risk assessment in medicinal materials [9]. High-throughput sequencing (HTS) provides an effective means of characterizing fungal community dynamics during the storage of medicinal herbs. For instance, fungal microbiome analyses have been performed on *Arecae semen*, *Cassiae semen*, and *Magnolia officinalis* to reveal how sampling locations, processing methods, and storage environments affect microbial diversity and toxin production [10,11,12].

To mitigate aflatoxin contamination, chemical preservatives have been extensively tested, but their potential toxicity, residue accumulation, and environmental concerns limit large-scale application. In this context, plant essential oils (EOs), known for their broad-spectrum antifungal activity, low toxicity, and environmental safety, have emerged as promising natural alternatives. Peppermint essential oil (PEO), in particular, has been reported to effectively suppress fungal growth and toxin production in fruits, vegetables, grains, and animal feed [13,14,15,16,17,18]. PEO contains major constituents such as menthol, isomenthone, and D-limonene, and has been reported to effectively inhibit the growth of *A. flavus* and reduce aflatoxin production [19,20,21]. However, its potential in preventing aflatoxin contamination in Chinese herbal medicines, especially in Polygalae Radix, remains largely unexplored.

Therefore, it is necessary to investigate the dynamic succession of fungal communities in Polygalae Radix during storage and to evaluate the feasibility of natural antifungal agents for mycotoxin prevention. Addressing these research gaps is essential for advancing post-harvest safety management. Such work will deepen our understanding of fungal and herb interactions during storage and provide a scientific foundation for developing natural, safe, and effective antifungal preservation strategies for Chinese medicinal materials.

## 2. Results

### 2.1. Fungal Community Changes During the Storage of Polygalae Radix Samples

#### 2.1.1. Diversity of Fungal Communities in Polygalae Radix Samples

**Figure 1:**
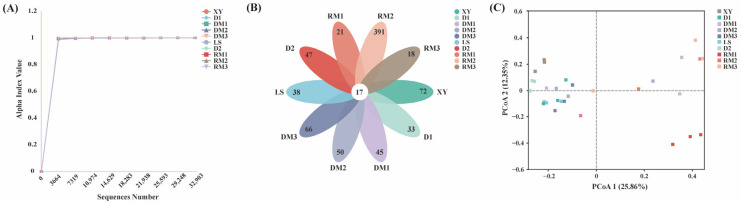
Analysis of fungal community diversity and abundance. (**A**) Rarefaction curves of all samples. (**B**) Petal diagram of shared and unique ASVs of all samples. (**C**) PCoA analysis based on Bray–Curtis distance.

A total of 1067 Amplicon Sequence Variants (ASVs) were detected after the deletion of chimeric sequences. The Good’s coverage for the observed ASVs of fungal communities exceeded 0.99 for all samples, and the rarefaction curve tended to flatten with the increasing number of sequences, indicating that the sequencing depth of all samples was sufficient to capture the fungal community diversity (Figure 1A). Eighteen ASVs were shared by ten samples of Polygalae Radix (Figure 1B). RM3 exhibited the highest number of unique ASVs, with 392. Chao1 and Shannon indices were utilized to assess fungal community richness and diversity in each sample (Table 1). The PCoA results showed that samples DM1, RM1, and RM3 exhibited significant differences in fungal community composition compared with other samples (Figure 1C), suggesting that the storage process has a pronounced impact on the fungal community structure of Polygalae Radix.

#### 2.1.2. Composition of Fungal Communities in Polygalae Radix Samples

**Figure 2:**
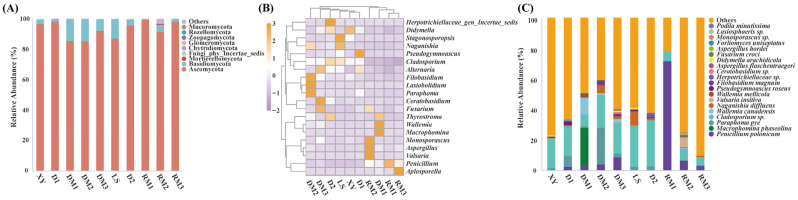
Fungal community composition analyses. (**A**) Phylum-level taxonomic composition of fungal community. (**B**) Heatmap of the relative abundance of the top 20 most abundant genera in all samples. (**C**) Composition of the top 20 most abundant fungal species at the species level.

Across the ten samples, a total of nine fungal phyla, 32 classes, 62 orders, 127 families, 211 genera, and 263 species were identified. At the phylum level, *Ascomycota* was predominant, accounting for 85.33–98.16% of fungal reads in all samples, while other detected phyla and unclassified ASVs were present at low relative abundance (Figure 2). At the genus level, *Penicillium*, *Paraphoma*, *Didymella*, and *Cladosporium* were dominant taxa. Based on genus-level composition, samples DM1, RM1, RM2, and RM3 clustered together, with the relative abundance of *Penicillium* markedly elevated in these samples, indicating that storage duration strongly affected the fungal community structure of Polygalae Radix. The genus *Wallemia* was detected in DM1, DM2, and DM3 with relative abundances exceeding 1%, whereas it was absent from freshly harvested samples. At the species level, *Penicillium polonicum* was the most abundant species within the genus *Penicillium*. Its relative abundance was below 2.19% in XY, LS, and D2, but exceeded 2.79% in all six long-term storage samples, reaching as high as 71.52% in RM1.

**Figure 3:**
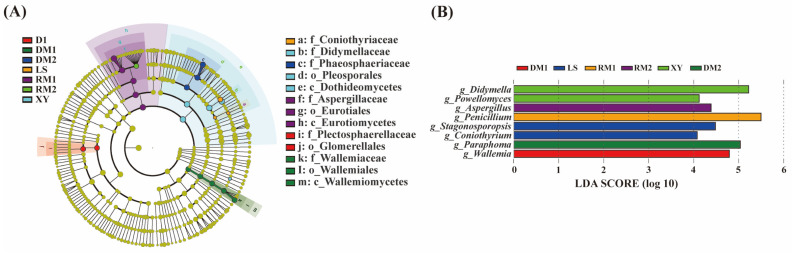
Linear discriminant analysis. (**A**) Significantly enriched fungal taxa showed by cladograms based on LEfSe analysis. (**B**) Scores for the significantly enriched fungal genera showed by bar chart based on LEfSe analysis.

The fungal community composition of Polygalae Radix in different samples was further investigated using Linear discriminant analysis effect Size (LEfSe). *Didymella* and *Powellomyces* were significantly enriched in the freshly harvested sample XY (Figure 3). In the freshly harvested sample LS, the relative abundances of *Stagonosporopsis* and *Coniothyrium* were the highest. *Wallemia* and *Paraphoma* were significantly enriched in DM1 and DM2, respectively. Notably, *Penicillium* and *Aspergillus*, two common toxigenic genera in medicinal materials, were identified as biomarkers of RM1 and RM2, respectively.

#### 2.1.3. Functional Prediction

**Figure 4:**
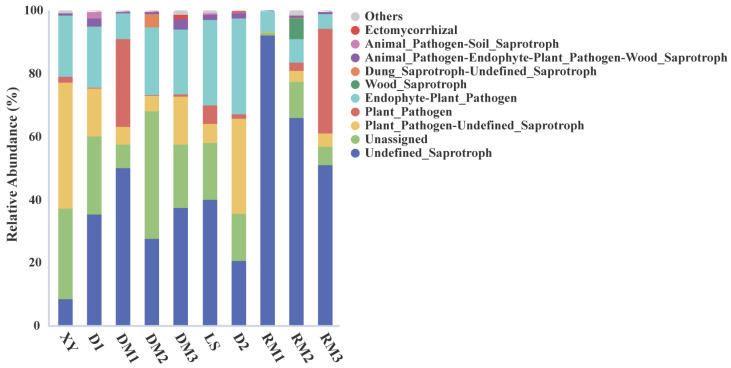
The top 10 functional groups of fungi inferred from the program FUNGuild.

Based on FUNGuild analysis, saprotrophic fungi dominated the fungal community in samples stored for more than one month (Figure 4). The relative abundances of undefined saprotrophs reached 92.02%, 65.91%, and 50.73% in RM1, RM2, and RM3, respectively. Plant pathogenic fungi showed a marked increase in DM1 and RM3. In conclusion, storage for more than one month significantly increased the potential risk of contamination of Polygalae Radix by plant pathogenic and saprotrophic fungi.

#### 2.1.4. Analysis of Mycotoxigenic Fungi of All Samples

**Figure 5:**
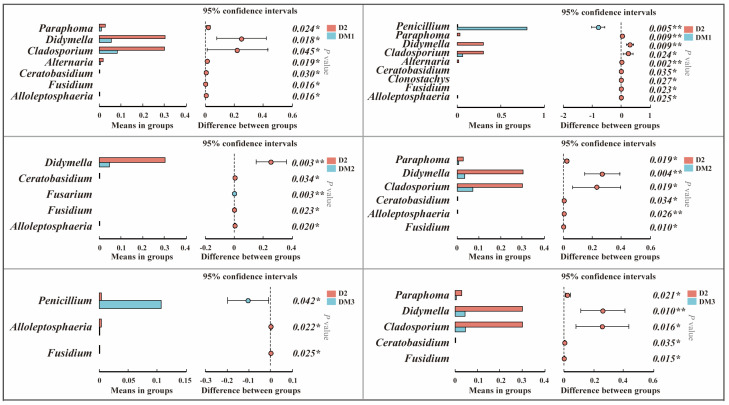
Wilcoxon rank-Sum test of genus-level differences between sample D2 and long-term stored samples. Significance levels are denoted as: * *p*  <  0.05, ** *p*  <  0.01.

Using sample D2 (air-dried for two days) as a reference, the Wilcoxon rank-sum test was applied to analyze differences in fungal communities at the genus level between long-term stored samples and D2. The results demonstrated significant alterations in fungal community composition following prolonged storage (Figure 5). In most of the long-term stored samples, the relative abundances of *Paraphoma*, *Didymella*, and *Cladosporium* were significantly reduced compared with D2 (*p* < 0.05). A significant increase in the relative abundance of *Fusarium* was observed in DM2, while the relative abundance of *Penicillium* was significantly elevated in DM3 and RM1 (*p* < 0.05).

**Figure 6:**
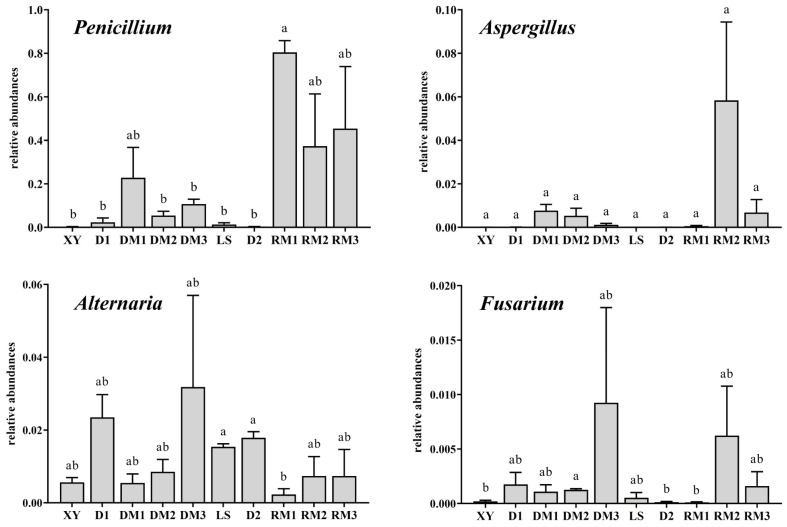
The relative abundances of common toxigenic fungal genera in all samples. Different lowercase letters indicate significant differences (*p* < 0.05).

Marked differences were observed in the relative abundances of potentially toxigenic genera (including *Penicillium*, *Alternaria*, *Fusarium*, and *Aspergillus*) between long-term stored samples and D2. Compared with D2, the relative abundance of *Penicillium* sharply increased and became the predominant genus in long-term stored samples, while *Aspergillus* also showed elevated relative abundances (Figure 6). The relative abundances of *Penicillium* were 22.81%, 5.39%, 10.72%, 80.43%, 37.32%, and 45.35% in DM1, DM2, DM3, RM1, RM2, and RM3, respectively, whereas in the other samples the values ranged only from 0.28% to 2.33%. The relative abundance of *Fusarium* was elevated in long-term stored samples compared with the other samples, with a significant increase observed in DM2 after two months of storage (*p* < 0.05). *Aspergillus* was either absent or detected at extremely low relative abundances in fresh samples (XY, LS) and in samples air-dried for only two days (D1, D2). However, after 1–3 months of storage, *Aspergillus* displayed varying levels of increase, reaching up to 5.84% in RM2. A total of thirteen *Aspergillus* species were identified across all samples, including the aflatoxigenic species *A. flavus* detected in RM2, suggesting that the middle to late storage stage represents a critical period for the enrichment of *A. flavus*. Collectively, these findings indicate that prolonged storage promotes the proliferation of toxigenic fungi, thereby increasing the potential risk of mycotoxin contamination in Polygalae Radix.

### 2.2. Antifungal Effects of PEO Against A. flavus in Stored Polygalae Radix

#### 2.2.1. Growth Status and Enumeration of *A. flavus* in Polygalae Radix

**Figure 7:**
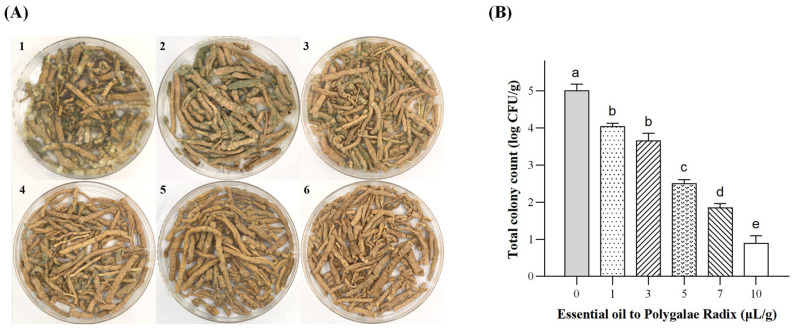
Effects of different PEO dosages on *A. flavus* growth in Polygalae Radix. (**A**) Growth status of *A. flavus* in each treatment group (1: control group without PEO; 2–6: 1, 3, 5, 7, and 10 μL/g PEO, respectively). (**B**) Log-transformed total colony counts of *A. flavus* in each treatment group. Different lowercase letters indicate significant differences. Data were analyzed by one-way ANOVA followed by Tukey’s multiple comparison test. Different lowercase letters indicate significant differences (*p* < 0.05).

The GC-MS analysis showed that the principal antifungal constituents in the essential oil were D-limonene and menthone, which accounted for 11.88% and 5.13%, respectively. Different volumes of PEO were added to an equal mass of Polygalae Radix, and the treated herb samples were incubated under sealed conditions for 30 days at 28–30 °C and 85–90% relative humidity, which are favorable for *A. flavus* growth. Both visual observation (Figure 7A) and plate counting (Figure 7B) indicated that PEO exerted a clear dose-dependent inhibitory effect on mold proliferation. In the control group without PEO, the herb surface was heavily colonized by dense mycelia and conspicuous yellow-green spore masses, displaying typical signs of *A. flavus* contamination. In contrast, increasing PEO concentrations gradually suppressed fungal growth. At 1 μL/g, mold growth was suppressed but numerous dark green colonies and spores persisted. With 3–7 μL/g, mycelial coverage decreased markedly, and the colony color shifted from dark green to grayish-white, suggesting metabolic inhibition. At the highest concentration (10 μL/g), no visible mycelia or spores were detected, and the herb surface remained intact without apparent contamination.

Quantitative analysis further confirmed these observations: colony counts declined from 5.01 log CFU/g in the control to 4.05, 3.67, 2.51, 1.86, and 0.91 log CFU/g at 1, 3, 5, 7, and 10 μL/g, corresponding to reductions of approximately 19%, 27%, 50%, 63%, and 82%, respectively (*p* < 0.05) (Figure 7B).

#### 2.2.2. Determination of Aflatoxin Content in Polygalae Radix by IAC-HPLC-FLD

**Figure 8:**
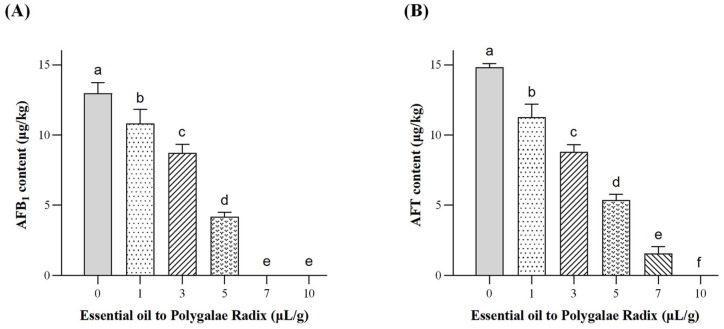
Effects of different PEO dosages on AFB_1_ and AFT levels in Polygalae Radix. (**A**) AFB_1_ content in each treatment group. (**B**) AFT content in each treatment group. Different lowercase letters indicate significant differences. Data were analyzed by one-way ANOVA followed by Tukey’s multiple comparison test. Different lowercase letters indicate significant differences (*p* < 0.05).

PEO treatment significantly reduced aflatoxin accumulation in Polygalae Radix in a concentration-dependent manner (Figure 8). In the control group, AFB_1_ and AFT contents reached 12.98 μg/kg and 14.84 μg/kg, respectively, consistent with active fungal growth and toxin synthesis. At low concentrations (1 and 3 μL/g), AFB_1_ levels decreased to 10.84 and 8.73 μg/kg, representing reductions of 17% and 33%, while AFT declined by 24% and 41% compared with the control. At 5 μL/g, AFB_1_ and AFT were reduced by 68% and 64%, respectively, indicating marked inhibition of toxin biosynthesis. A stronger effect was observed at 7 μL/g, where AFB_1_ was reduced to undetectable levels and AFT decreased by nearly 90%. At the highest concentration (10 μL/g), neither AFB_1_ nor AFT were detectable, demonstrating that PEO at this dosage effectively suppressed aflatoxin production in Polygalae Radix during storage.

## 3. Discussion

### 3.1. Changes in Fungal Communities During the Storage of Polygalae Radix

The results of this study indicated that, compared with short-term storage, prolonged storage significantly altered the diversity and structure of the Polygalae Radix fungal community, resulting in an increased abundance of various saprophytic and toxigenic fungi. In the DM1 sample, the biomarker was *Wallemia*, a xerophilic or halophilic genus capable of causing mold growth in low-moisture foods and producing food-borne mycotoxins [22,23]. *Wallemia sebi* was observed in all long-term stored samples except RM1. This species is known to cause cutaneous and subcutaneous infections in humans and can lead to respiratory disease, especially in individuals with occupational exposure such as farmers. Furthermore, it is capable of synthesizing mycotoxins, including walleminol, walleminone, and wallimidione [24,25]. In addition, *Penicillium*, *Alternaria*, *Fusarium*, and *Aspergillus* are recognized as the major mycotoxin-producing genera in medicinal herbs, capable of generating aflatoxins, ochratoxins, fumonisins, zearalenone, and deoxynivalenol [26,27]. Among the six long-term stored samples, *Penicillium polonicum* was notably enriched. This species is capable of producing the neurotoxic compound verrucosidin as well as ochratoxin A (OTA), both of which pose potential risks to the nervous system and hepatorenal function upon long-term exposure [28,29,30]. Furthermore, *A*. *flavus*, a known aflatoxin producer, was identified in the RM2 sample after two months of storage, highlighting the potential risk of aflatoxin contamination in Polygalae Radix. If the herbs were already contaminated with *A. flavus* prior to storage, toxin levels could rise sharply.

Chang et al. [31] investigated Polygalae Radix after six months of storage and found that 33.3% of the samples exceeded the limits for AFB_1_ and AFT specified in the Pharmacopoeia of the People’s Republic of China. Similarly, Zhang et al. [32] observed a significant increase in *Cladosporium* and *Fusarium* during post-harvest processing of Polygalae Radix, and with prolonged storage duration, the growth of toxigenic fungi and the potential risk of aflatoxin contamination progressively increased. Similar phenomena have also been reported in the storage of other medicinal herbs [33]. These findings are consistent with our observations, indicating that long-term storage promotes fungal growth and increases the risk of contamination by aflatoxins and other mycotoxins.

Comparison of samples from two production regions, Yulin City (Shaanxi Province) and Yuncheng City (Shanxi Province), revealed distinct fungal community structures in Polygalae Radix. Considering that humidity and temperature are key environmental factors influencing fungal growth and colonization, these regional differences in community composition may be closely related to variations in humidity and temperature between the two sites, although further experimental validation is required.

In conclusion, dynamic analysis of Polygalae Radix during 1–3 months of storage revealed that storage beyond one month favored the growth of saprophytic and toxigenic fungi. Consequently, substantially increased the risk of aflatoxin and other mycotoxin contamination.

### 3.2. Inhibitory Effects of PEO on A. flavus and Its Potential Application in the Storage of Traditional Chinese Herbal Medicines

In this study, colony counting and HPLC quantification confirmed that PEO fumigation significant inhibitory effects against *A. flavus* in Polygalae Radix, exhibiting a clear dose-dependent response. At low doses (1–3 μL/g), PEO partially suppressed mycelial growth but failed to reduce aflatoxin levels below pharmacopoeial limits, indicating insufficient safety. When the dose was increased to 5 μL/g, AFB_1_ levels approached the limit but the risk remained. In the 7 μL/g treatment group, inhibition was more pronounced; however, trace amounts of mycelium remained, potentially leading to recurrent contamination. Only at a dose of 10 μL/g was mycelial growth completely inhibited on the herb surface, and both AFB_1_ and AFT were undetectable, indicating that this is the critical dose for effective preservation. It is worth noting that the small residual colony count observed under the highest PEO treatment may be attributed to the high stress resistance of persistent A. flavus conidia or the recovery of a small fraction of sub-lethally injured spores.

Although the precise antifungal mechanism of PEO has not been fully elucidated, previous studies have demonstrated that PEO and other plant essential oils (EOs) can inhibit fungal growth and toxin production through multiple pathways. At the cellular level, PEO alters hyphal morphology and compromises the integrity of both the cell wall and membrane [34]. At the subcellular level, it modifies membrane lipid composition and metabolic products, resulting in intracellular leakage and disruption of the antioxidant system, thereby aggravating cellular injury [35]. At the molecular level, numerous studies have demonstrated that certain essential oil constituents can effectively inhibit the growth of A. flavus and reduce mycotoxin production. For instance, molecular docking studies revealed that D-limonene exhibits strong binding energies with three key regulatory enzymes of A. flavus, indicating its potential to suppress their activity and consequently reduce AFB_1_ production [36]. In addition, the essential oil from Hesperozygis marifolia, whose major components are (R)-pulegone (40.75%), isomenthone (30.34%) and menthone (4.46%), was reported to completely inhibit the growth of A. flavus at a concentration of 2.0 mg/mL [37]. Moreover, essential oils can broadly inhibit fungi by suppressing sporulation, interfering with mitochondrial function, and inducing denaturation or apoptosis/necrosis of cellular components [38,39,40]. Notably, the natural volatility of essential oils allows rapid penetration into fungal structures and uniform diffusion in a closed system, providing a practical advantage for storage applications [41]. These mechanisms offer a reasonable explanation for the observed reductions in fungal load and aflatoxin levels in Polygalae Radix in this study.

It should also be noted that the A. flavus strain used in this study was capable of producing both B- and G-type aflatoxins, a toxigenic profile that, although once considered rare, has been increasingly documented in natural isolates [42,43,44,45]. This finding suggests that our strain may represent an underrecognized yet biologically significant toxigenic type. Importantly, this observation does not affect the major conclusions of the present study, which emphasize the elevated risk of aflatoxin contamination during the storage of Polygalae Radix and the potent inhibitory effects of PEO on fungal growth and aflatoxin accumulation.

In conclusion, PEO effectively inhibits *A. flavus* growth and toxin production via multiple mechanisms and, compared to chemical preservatives, offers advantages such as natural origin, environmental friendliness, and low residue risk. The present results indicate that a PEO treatment of 10 μL/g can effectively suppress mold proliferation and aflatoxin accumulation in Polygalae Radix under the experimental conditions, providing a feasible natural strategy for safe storage. Given its multifaceted antifungal mechanisms and favorable safety profile, PEO shows considerable potential as a natural preservative for high-risk medicinal herbs. Future research should focus on the stability of its vapor phase, compatibility with packaging materials, and sustained efficacy under complex storage conditions to promote standardized applications in the preservation of medicinal herbs and food products. In addition, given the increasing dominance of Penicillium during storage, future studies will also investigate the inhibitory effects of PEO on OTA production and other mycotoxins beyond AFB_1_. Such research will support the development of a more integrated safety control strategy for high-risk medicinal materials.

This study provides novel insights into the safety control of stored medicinal herbs from both theoretical and practical perspectives. Theoretically, our high-throughput sequencing analysis delineates a temporal succession of the fungal microbiome in Polygalae Radix during storage. We identified that storage beyond one month constitutes a critical period for the marked enrichment of saprophytic and toxigenic fungi, including *Aspergillus* and *Penicillium* genera. This discovery delineates a critical time window for targeted antifungal intervention. Practically, we introduce and validate an effective, eco-friendly antifungal strategy—vapor-phase fumigation with peppermint essential oil (PEO). More importantly, we determined that a concentration of 10 μL/g PEO is the critical dose required to completely inhibit *A. flavus* growth and prevent aflatoxin accumulation without compromising herb quality. This specific parameter provides an actionable reference for industrial application. By integrating microbiome analysis with the validation of a natural antifungal agent, this research establishes a translatable framework for developing science-based, safe, and sustainable preservation protocols for high-risk medicinal herbs and other agricultural products.

## 4. Conclusions

This study revealed the dynamic changes in the fungal community structure of Polygalae Radix during storage and demonstrated that prolonged storage markedly promotes the proliferation of saprophytic and toxigenic fungi, thereby increasing the potential risk of aflatoxin contamination. To address this issue, fumigation experiments with PEO showed that treatment at 10 μL/g effectively suppressed the growth and reproduction of *A. flavus* in Polygalae Radix and significantly reduced aflatoxin levels in the samples (*p* < 0.05). Accordingly, 10 μL/g can be considered a reference concentration for vapor-phase antifungal application during the storage of medicinal herbs. These findings provide both a scientific basis and practical guidance for ensuring the quality and safety of herbal medicines.

## 5. Materials and Methods

### 5.1. Materials

Polygalae Radix: To examine fungal community dynamics during Polygalae Radix storage, three-year-old roots were collected from Yulin City (Shaanxi Province) and Yuncheng City (Shanxi Province). The plant material was authenticated by Dr. Jianping Han of the Institute of Medicinal Plant Development, Chinese Academy of Medical Sciences, as *Polygala tenuifolia* Willd. After collection, all samples were air-dried for two days and then stored for the duration of the experiment in a cool, shaded environment maintained at room temperature (approximately 25 °C) and a relative humidity of 50–60%. For each origin, samples were analyzed at five time points (fresh, 2 days, 1 month, 2 months, and 3 months) to monitor temporal dynamics. Each of these 10 experimental groups consisted of three independent biological replicates, making a total of 30 samples for sequencing analysis. All samples were immediately frozen at −80 °C until DNA extraction and sequencing (Table 2). Separately, to evaluate the antifungal activity of PEO against *A. flavus*, commercially available Polygalae Radix samples were obtained from the Anguo Herbal Medicine Market (Anguo, China) and subjected to fumigation experiments.

*A. flavus* isolate: The *A. flavus* isolate used in this study (designated as GLJ-AF01) was originally isolated from moldy cereal crops. It was kindly provided by the Standard and Quality Center of the National Food and Strategic Reserves Administration.

### 5.2. Methods

#### 5.2.1. Fungal Community Dynamics During Polygalae Radix Storage

##### DNA Extraction and Sequencing

Total genomic DNA from all Polygalae Radix samples was extracted using CTAB method. The ITS sequence was amplified with ITS1F (5’-CTTGGTCATTTAGAGGAAGTA-3’)/ITS2R (5’-GCTGCGTTCTTCATCGATG-3’) primers [46,47]. Three replicates were performed for each sample. The Polymerase Chain Reaction (PCR) system is composed as follows: 15 μL of Phusion^®^ High-Fidelity PCR Master Mix (New England Biolabs, Ipswich, MA, USA), 0.2 μM of forward and reverse primers, and about 10 ng template DNA. Thermal cycling consisted of initial denaturation at 98 °C for 1 min, followed by 30 cycles of denaturation at 98 °C for 10 s, annealing at 50 °C for 30 s, elongation at 72 °C for 30 s, and final extension at 72 °C for 5 min. The integrity and concentration of PCR products were verified by 2% agarose gel electrophoresis. PCR products were purified with Qiagen Gel Extraction Kit (Qiagen, Hilden, North-Rhine Westphalia, Germany). Sequencing libraries were generated using TruSeq^®^ DNA PCR-Free Sample Preparation Kit (Illumina, San Diego, CA, USA) and index codes were added. The library quality was assessed on the Qubit 2.0 Fluorometer (Thermo Fisher Scientific, Waltham, MA, USA) and Agilent Bioanalyzer 2100 system. Finally, the library was sequenced on an Illumina NovaSeq platform by Novogene Co., Ltd. (Beijing, China) and 250 bp paired-end reads were generated.

##### Bioinformatics Analysis

Paired-end reads were assigned to samples based on their unique barcode and truncated by cutting off the barcode and primer sequence. The resulting reads were then merged using FLASH. Quality filtering on the raw tags was performed using the Fastp v0.23.1 software to obtain high-quality Clean Tags. The tags were compared with the UNITE database (https://unite.ut.ee/ (accessed on 5 September 2024)) using UCHIME algorithm (http://www.drive5.com/usearch/manual/uchime_algo.html (accessed on 5 September 2024)) to detect chimera sequences, and then the chimera sequences were removed. The final set of high-quality reads, referred to as Effective Tags, was obtained. Denoising was performed using the DADA2 module in QIIME2 software (Version 202202) to obtain initial ASVs (Amplicon Sequence Variants). Species annotation was performed using QIIME2 software and the annotation database is Unite Database. To study phylogenetic relationship of each ASV and the differences in the predominant species among different samples (groups), multiple sequence alignment was performed using QIIME2 software. The absolute abundance of ASVs was normalized using a standard of sequence number corresponding to the sample with the least sequences. Subsequent analysis of alpha diversity and beta diversity were all performed based on the output normalized data.

Petal diagrams were produced in R to identify the shared and unique ASVs among different samples. Principal coordinate analysis (PCoA) of fungal community composition was performed using Bray–Curtis distance. The fungal community’s relative abundance distribution histogram across different classification levels for all samples is generated using SVG function in Perl. Biomarkers were identified by the Linear discriminant analysis effect size (LEfSe) with a linear discriminant analysis (LDA) score > 4.0 and *p* < 0.05. Fungal guilds were predicted using FUNGuild(https://www.funguild.org/ (accessed on 5 September 2024)). The Wilcoxon rank-sum test was used to compare fungal composition between the two samples. The active component contents and relative abundance of common mycotoxin-producing fungi were visualized as histograms using GraphPad Prism v10.0.3, and the statistical differences were analyzed using one-way analysis of variance (ANOVA) with SPSS V26.0 (IBM, Armonk, New York, NY, USA).

#### 5.2.2. Evaluation of the Optimal Concentration of PEO Against *A. flavus* in Stored Polygalae Radix

To evaluate the optimal antifungal concentration of PEO against *A. flavus* in stored Polygalae Radix, commercially obtained Polygalae Radix was used together with *A. flavus* as the test isolate. PEO (Lot No. F60015) was purchased from Shandong Yousuo Chemical Technology Co., Ltd. (Linyi City, Shandong Province, China). The identity of the strain was verified by molecular analysis through PCR amplification of the ITS region using universal primers ITS1F (5’-CTTGGTCATTTAGACGAAGTAA-3’) and ITS4 (5’-TCCTCCGCTTATTGATATGC-3’). PCR products were sequenced, and the resulting sequences were compared with reference data in the NCBI GenBank database to confirm species identity.

##### Preparation of *A. flavus* Spore Suspension

The activated *A. flavus* isolate was cultured on potato dextrose agar (PDA) (Lot: GC-02-023, Aoboxbio-Technology Co., Ltd., Shanghai, China) plates at 30 °C for 7 days to obtain mature spores. Following incubation, 5 mL of 0.05% Tween 80 solution was gently added to each plate and allowed to stand until the colony surface was fully moistened. The resulting spore suspension was collected into sterile test tubes, with the tube openings covered by sterile cotton plugs to remove mycelial fragments. The elution step was repeated twice on the same plate to ensure sufficient spore recovery. After removal of the cotton plugs, the suspension was centrifuged at 8500 rpm for 15 min at 4 °C. The supernatant was discarded. The pellet was resuspended in 20 mL of phosphate-buffered saline (PBS), vortexed for 30 s, and centrifuged again under the same conditions, followed by removal of the supernatant. The final spore concentration was determined using the plate count method and adjusted to 10^6^ CFU/mL with PBS.

##### Inoculation of Polygalae Radix with *A. flavus* Spores

All Polygalae Radix samples were sterilized by UV-C irradiation (254 nm, 8 h, 20 cm distance) [48], and the moisture content was adjusted to 20% using double-distilled water [49]. Ten grams of each sterilized sample were randomly weighed, placed in a 90 mm Petri dish, and evenly spread in a single layer. All procedures were conducted under aseptic conditions. Subsequently, 10 µL of *A. flavus* spore suspension (10^6^ CFU/mL) was inoculated at the center of each dish.

##### Vapor-Phase Fumigation with PEO

Prior to the antifungal assay, the chemical composition of the PEO was analyzed by gas chromatography–mass spectrometry (GC–MS) using a Trace ISQ system (Thermo Fisher Scientific, USA), according to the method of Skalicka-Woźniak K [50].

The filter paper disk diffusion method was used to evaluate the inhibitory effect of PEO on the growth of *A. flavus* in Polygalae Radix samples. Six treatment groups were prepared by applying different volumes of PEO (0, 10, 30, 50, 70, and 100 µL) onto sterile 6 mm-diameter filter paper disks. The disks were affixed to the inner surface of the lid of Petri dishes containing 10 g of Polygalae Radix material. The filter paper ensured no direct contact with the herbal samples, so that the essential oil acted exclusively through vapor-phase fumigation. The corresponding essential oil-to-herbal material mass ratios were 0, 1, 3, 5, 7, and 10 µL/g, with three replicates per group. The Petri dishes were sealed with parafilm and incubated at room temperature for 30 days.

##### Assessment of *A. flavus* Growth

Mold Enumeration: After 30 days of treatment, *A. flavus* colonies in each treatment group was enumerated following the procedures outlined in National Food Safety Standard of the People’s Republic of China: Food Microbiological Examination-Enumeration of Molds and Yeasts (GB 4789.15-2016) [51].

Aflatoxin Assay: After 30 days of treatment, the aflatoxin content in Polygalae Radix samples from each treatment group was determined using immunoaffinity chromatography-high-performance liquid chromatography-fluorescence detection (IAC-HPLC-FLD) [52,53,54]. The herbal material was ground into powder, passed through a No. 2 sieve (24 mesh), and stored for further analysis. A 0.5 g aliquot of the powdered sample was accurately weighed into a centrifuge tube, mixed with 1 g NaCl, and extracted with 25 mL of methanol-water (70:30, *v*/*v*; methanol, Lot: M116125, Shanghai Aladdin Biochemical Technology Co., Ltd., China) by ultrasonication for 15 min. The extract was centrifuged at 10,000 rpm for 5 min, and 5 mL of the supernatant was diluted to 50 mL with ultrapure water. The diluted extract was loaded onto an immunoaffinity column (Pribolab, Zhengzhou, Henan Province, China) at a flow rate of approximately 3 mL/min, washed with 10 mL of water, and eluted with 2 mL of methanol. The eluate was collected, adjusted to a final volume of 2 mL, and filtered through a 0.22 μm membrane prior to chromatographic analysis. Aflatoxin mixed standard (Pribolab, China) was serially diluted with methanol-water (70:30, *v*/*v*) to prepare working solutions.

Chromatographic analysis was performed on the LC-2030 HPLC system (Shimadzu, Japan) equipped with the Agilent ZORBAX Extend-C18 reversed-phase column (5 μm, 4.6 mm × 250 mm, Agilent, Santa Clara, CA, USA). The mobile phase consisted of methanol-water (40:60, *v*/*v*), delivered isocratically at a flow rate of 1.0 mL/min. Post-column photochemical derivatization was conducted at 254 nm, and detection was achieved with a fluorescence detector set at an excitation wavelength of 360 nm and an emission wavelength of 450 nm. The column temperature was maintained at 30 °C, and the injection volume was 10 μL. Peak areas were recorded under these conditions, and aflatoxin concentrations were quantified using calibration curves generated from the standard solutions.

## Figures and Tables

**Table 1:** Alpha diversity indices.

Sample Name	Good’s Coverage Index	Chao1 Index	Shannon Index
XY	0.99978	95.28 ± 18.84 ^a^	2.94 ± 0.28 ^a^
D1	0.99988	68.75 ± 12.14 ^a^	2.87 ± 0.32 ^a^
DM1	0.99983	76.7 ± 15.25 ^a^	2.58 ± 0.86 ^a^
DM2	0.99991	72.17 ± 14.22 ^a^	2.99 ± 1.12 ^a^
DM3	0.99985	84.08 ± 46.19 ^a^	3.15 ± 0.63 ^a^
LS	0.99984	75.92 ± 11.16 ^a^	2.80 ± 0.38 ^a^
D2	0.99985	89.17 ± 13.04 ^a^	2.91 ± 0.24 ^a^
RM1	0.99997	33.33 ± 4.51 ^a^	1.47 ± 0.52 ^a^
RM2	0.99974	190.67 ± 203.32 ^a^	3.52 ± 1.88 ^a^
RM3	0.99992	51.58 ± 42.49 ^a^	1.49 ± 2.10 ^a^

Different lowercase letters indicate significant differences (*p* < 0.05).

**Table 2:** Information on samples used for fungal community analysis.

No.	Sample Name	Collection Location	Storage Conditions
1	XY	Yulin, Shaanxi	Freshly harvested samples
2	D1	Yulin, Shaanxi	Air-dried for 2 days
3	DM1	Yulin, Shaanxi	Stored for 1 month
4	DM2	Yulin, Shaanxi	Stored for 2 months
5	DM3	Yulin, Shaanxi	Stored for 3 months
6	LS	Yuncheng, Shanxi	Freshly harvested samples
7	D2	Yuncheng, Shanxi	Air-dried for 2 days
8	RM1	Yuncheng, Shanxi	Stored for 1 month
9	RM2	Yuncheng, Shanxi	Stored for 2 months
10	RM3	Yuncheng, Shanxi	Stored for 3 months

## Data Availability

The original contributions presented in this study are included in the article/Supplementary Materials. Further inquiries can be directed to the corresponding author(s).

## Supplementary Materials

The following supporting information can be downloaded at: https://www.mdpi.com/article/10.3390/toxins17120585/s1, Figure S1: Calibration curves for aflatoxins; Figure S2: Chromatogram of standards and samples.; Figure S3: Effects of different PEO dosages on AFB_2_, AFG_1_ and AFG_2_ levels in Polygalae Radix.; Figure S4: GC-MS ion chromatograms and chemical structures of antimicrobial components in peppermint essential oil; Table S1: Complete dataset of all ASVs.
