# Supplementary material for: Study on the Dynamic Changes in Fungal Communities During the Storage of Polygalae Radix and the Antifungal Effects of Peppermint Essential Oil
Source: Toxins (Basel). 2025 Dec 6;17(12):585. doi: 10.3390/toxins17120585 (PMC12737776; doi:10.3390/toxins17120585)
Supplement: Supplementary file 1 [file toxins-17-00585-s001.zip › Supplementary_Figures.pdf]

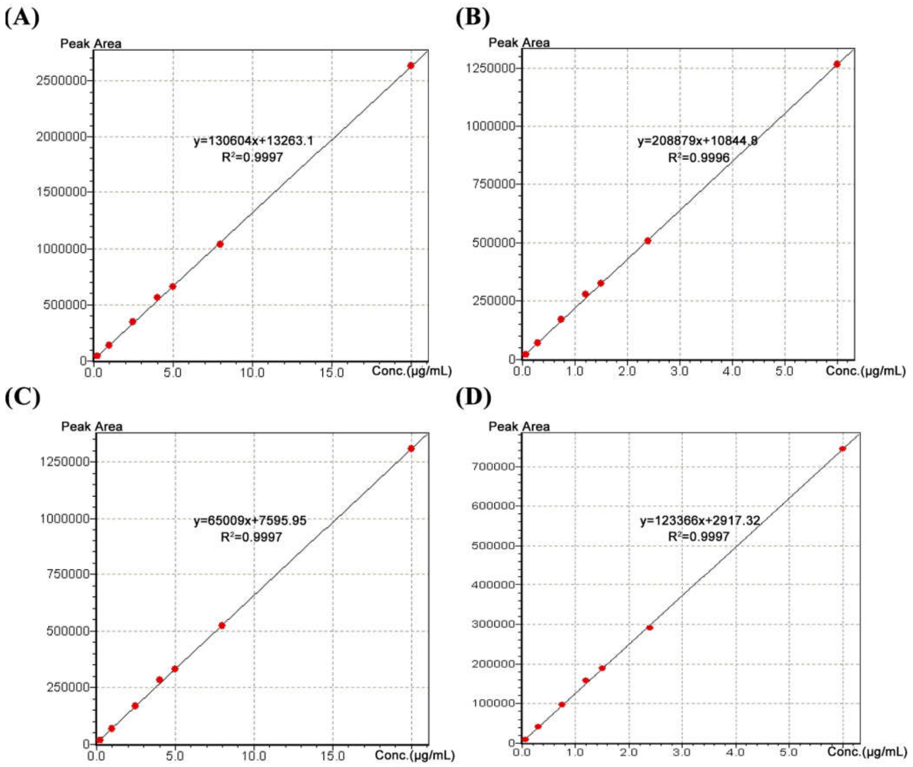

Figure S1 Calibration curves for aflatoxins. (A) AFB_1; (B) AFB_2; (C) AFG_1; (D) AFG_2.

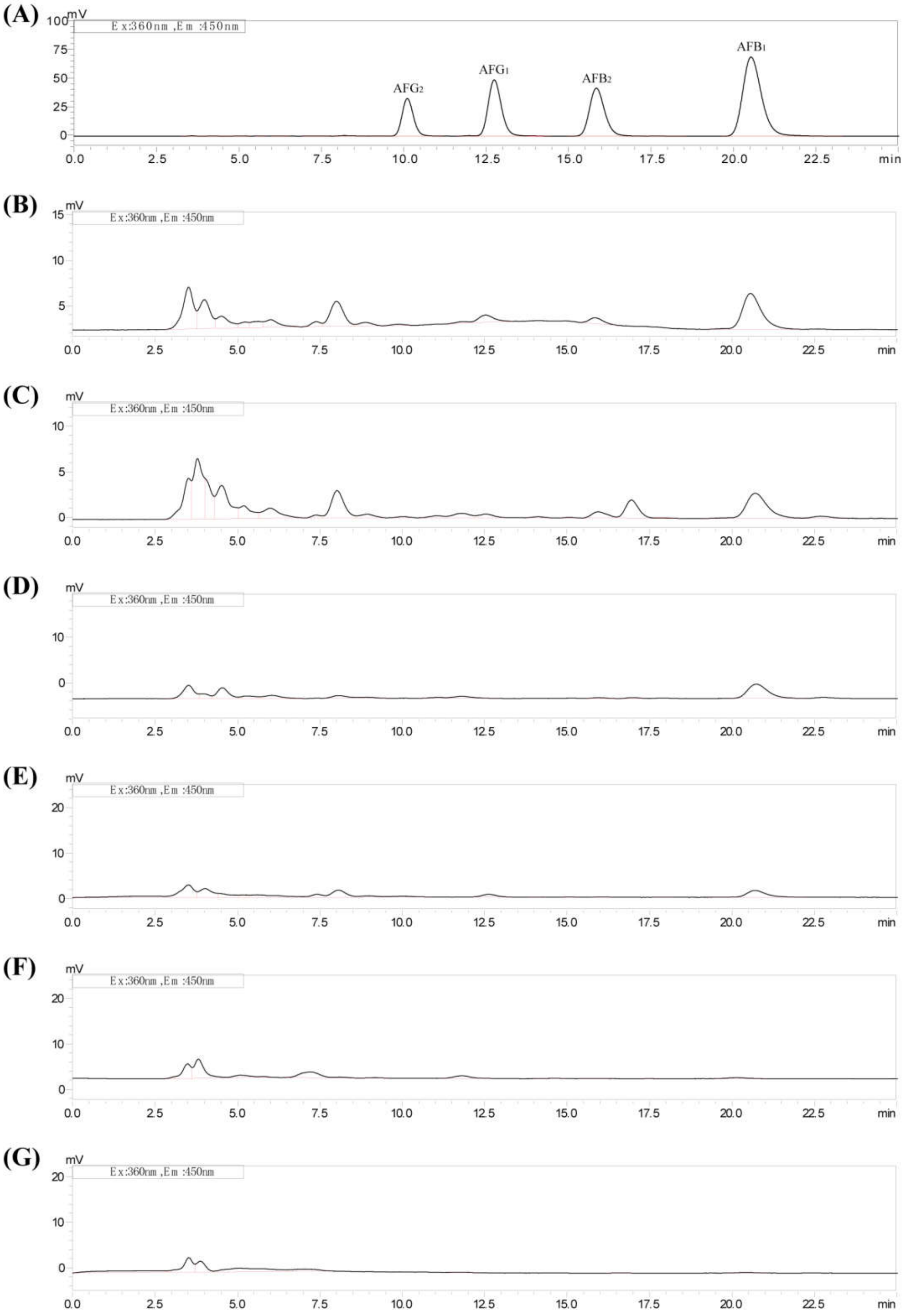

Figure S2 Chromatogram of standards and samples.(A) Aflatoxin standard; (B) Control sample without essential oil; (C–G) Samples treated with peppermint essential oil at concentrations of 1, 3, 5, 7, and 10 μL/g, respectively.

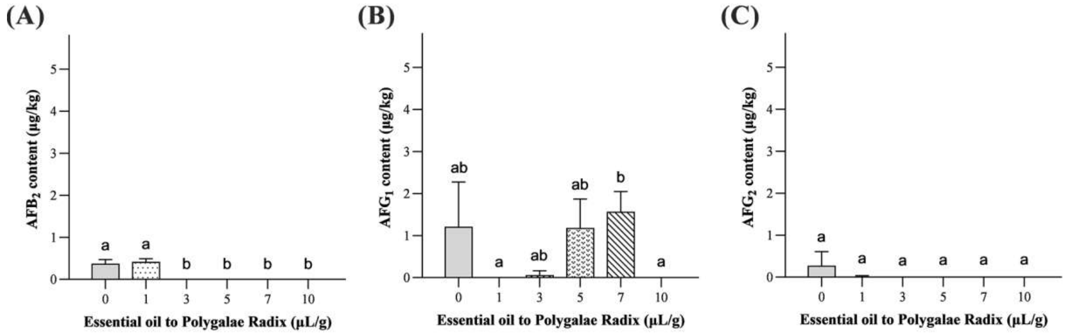

Figure S3 Effects of different PEO dosages on AFB_2, AFG_1 and AFG_2 levels in Polygalae Radix. (A) AFB_2 content in each treatment group; (B) AFG_1 content in each treatment group; (C) AFG_2 content in each treatment group.*Data were analyzed by one-way ANOVA followed by Tukey's multiple comparison test. Different lowercase letters indicate significant differences ($p < 0.05$).

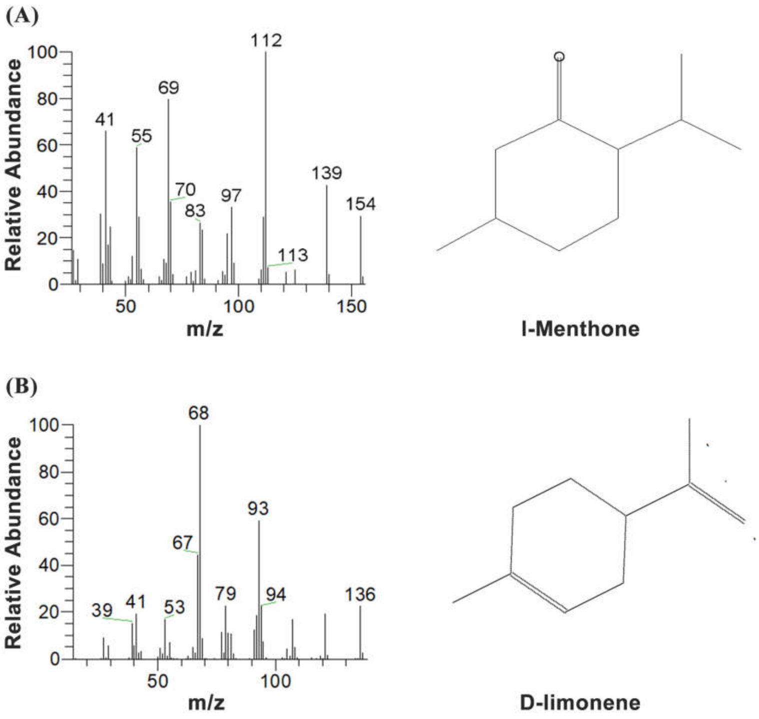

Figure S4 GC-MS ion chromatograms and chemical structures of antimicrobial components in peppermint essential oil. (A) Menthone; (B) D-Limonene.
